# Brain Histamine Is Crucial for Selective Serotonin Reuptake Inhibitors‘ Behavioral and Neurochemical Effects

**DOI:** 10.1093/ijnp/pyv045

**Published:** 2015-04-21

**Authors:** Leonardo Munari, Gustavo Provensi, Maria Beatrice Passani, Nicoletta Galeotti, Tommaso Cassano, Fernando Benetti, Renato Corradetti, Patrizio Blandina

**Affiliations:** Dipartimento di Neuroscienze, Psicologia, Area del Farmaco e Salute del Bambino, Sezione di Farmacologia e Tossicologia, Universitá di Firenze, Firenze, Italy (Drs Munari, Provensi, Passani, Galeotti, Benetti, Corradetti, and Blandina); Dipartimento di Medicina Clinica e Sperimentale, Universitá di Foggia, Viale Luigi Pinto, 1 - 71100, Foggia Italy (Dr Cassano); Centro de Memória-Instituto do Cérebro-Pontificia Universidade Católica do Rio Grande do Sul, Porto Alegre, Brazil (Dr Benetti).; Present address (L.M.): Department of Pharmacology and Systems Therapeutics, Icahn School of Medicine at Mount Sinai, New York, NY 10029.; Present address (F.B.): Departamento de Fisiologia, Instituto de Ciências Básicas da Saúde, Universidade Federal do Rio Grande do Sul, Rua Sarmento Leite 500, Porto Alegre, RS 90050-17, Brazil.

**Keywords:** Histidine decarboxylase, citalopram, reboxetin, CREB, tail suspension test, in vivo microdialysis

## Abstract

**Backgound:**

The neurobiological changes underlying depression resistant to treatments remain poorly understood, and failure to respond to selective serotonin reuptake inhibitors may result from abnormalities of neurotransmitter systems that excite serotonergic neurons, such as histamine.

**Methods:**

Using behavioral (tail suspension test) and neurochemical (in vivo microdialysis, Western-blot analysis) approaches, here we report that antidepressant responses to selective serotonin reuptake inhibitors (citalopram or paroxetine) are abolished in mice unable to synthesize histamine due to either targeted disruption of histidine decarboxylase gene (HDC^-/-^) or injection of alpha-fluoromethylhistidine, a suicide inhibitor of this enzyme.

**Results:**

In the tail suspension test, all classes of antidepressants tested reduced the immobility time of controls. Systemic reboxetine or imipramine reduced the immobility time of histamine-deprived mice as well, whereas selective serotonin reuptake inhibitors did not even though their serotonergic system is functional. In in vivo microdialysis experiments, citalopram significantly increased histamine extraneuronal levels in the cortex of freely moving mice, and methysergide, a serotonin 5-HT_1_/5-HT_2_ receptor antagonist, abolished this effect, thus suggesting the involvement of endogenous serotonin. CREB phosphorylation, which is implicated in the molecular mechanisms of antidepressant treatment, was abolished in histamine-deficient mice treated with citalopram. The CREB pathway is not impaired in HDC^-/-^ mice, as administration of 8-bromoadenosine 3’, 5’-cyclic monophosphate increased CREB phosphorylation, and in the tail suspension test it significantly reduced the time spent immobile by mice of both genotypes.

**Conclusions:**

Our results demonstrate that selective serotonin reuptake inhibitors selectively require the integrity of the brain histamine system to exert their preclinical responses.

## Introduction

Major depression is a common psychiatric disorder with a devastating socio-economic impact worldwide (Gustavsson et al., 2011). First-line treatments include selective serotonin reuptake inhibitors (SSRIs), yet fewer than 50% of patients respond adequately to medication (Berton and Nestler, 2006a). SSRI inefficacy may result from abnormalities of neurotransmitter systems that excite serotonergic neurons (Coplan et al., 2014). Histamine is synthesized from histidine by histidine-decarboxylase (HDC) (Haas et al., 2008) in neurons restricted to the hypothalamic tuberomamillary nucleus (TMN) and innervating most of the brain, including the raphe nuclei (Watanabe et al., 1983). Experimental studies demonstrated functional interactions between histaminergic and serotonergic systems (Airaksinen et al., 1989; Laitinen et al., 1995; Brown et al., 2002) that share control of functions impaired in depression, such as appetite, cognition, emotion, and sleep (Passani et al., 2004; Haas et al., 2008). Histamine H_1_-receptor activation increased the firing rate of serotonergic neurons (Eriksson et al., 2001). Interestingly, PET studies showed reduced H_1_-receptor density in the brain of depressed patients that positively correlated with the severity of clinical profile (Kano et al., 2004; Yanai and Tashiro, 2007). Here, we report that behavioral and neurochemical responses to SSRIs exclusively, and not to other antidepressants, are abolished in mice genetically or pharmacologically unable to synthesize histamine. Disruption of histamine neurotransmission affected not only behavioral responses but also the activation of intracellular pathways elicited by SSRIs. To this end, we used HDC^-/-^ mice, their HDC^+/+^ littermates, CD1 mice acutely deprived of histamine by injecting intracerebroventricular (i.c.v.) alpha-fluoromethylhistidine (α-FMHis), a suicide inhibitor of HDC (Garbarg et al., 1980), and sham-operated controls. Hence, using different experimental approaches, we demonstrate that histaminergic neurotransmission affects responses to SSRIs.

## Methods

### Chemicals

Citalopram hydrobromide (Tocris), reboxetine mesylate (Tocris), paroxetine hydrochloride hemihydrate (Sigma), imipramine hydrochloride (Sigma), and methysergide (Sigma) were freshly dissolved into saline (NaCl 0.9%) before use. Compounds were dissolved in a final volume of 10mL/kg. α-FMHis (synthesized at Abbott Laboratories, Chicago, IL) was injected i.c.v. at the dose of 5 µg dissolved in 5 µL of saline. All doses were calculated as mg/kg of the free base. Control animals received saline. In reverse dialysis experiments, drugs were diluted in the perfusing Ringer’s solution. All other reagents and solvents were of high performance liquid chromatography (HPLC) grade or the highest grade available (Sigma).

### Animals

All animals were housed in macrolon cages in temperature-controlled rooms (20–24°C), allowed free access to food and water, and kept on a 12-h-light/-dark cycle (light started at 7:00 am). Male CD-1 mice (25–30g body weight, Harlan, Italy) were used along with female and male inbred HDC^+/+^ and HDC^-/-^ mice of 11 to 13 weeks of age and 25 to 30mg body weight bred in the Centre for Laboratory Animals, Universitá di Firenze, Italy, and housed in a dedicated room. They were descendants of the 129/Sv mouse strain generated by Ohtsu (Ohtsu et al., 2001). Their genotype with regards to the HDC gene was determined according to the polymerase chain reaction (PCR) protocol described by Parmentier (Parmentier et al., 2002). All the experiments were performed in strict compliance with the EEC recommendations for the care and use of laboratory animals (2010/63/EU) and were approved by the Animal Care Committee of the Dipartimento di Neuroscienze, Psicologia, Area del Farmaco e Salute del Bambino, Sezione di Farmacologia e Tossicologia, Universitá di Firenze, Italy. Ethical policy of the University of Florence complies with the Guide for the Care and Use of Laboratory Animals of the US National Institutes of Health (NIH publication no. 85-23, revised 1996; University of Florence assurance number: A5278-01). Every effort was made to minimize animal suffering and to reduce the number of animals used. Animals were handled for at least 4 days before experiments begun to become acclimatized to human contact.

### Tail Suspension Test

tail suspension test (TST) was carried out as described (Steru et al., 1985). Briefly, 30 minutes after i.p. injection of saline or drugs, mice were individually suspended by the tail to a horizontal ring stand bar (distance from floor=30cm) using adhesive tape (distance from tip of tail=2cm). Typically, mice demonstrated several escape-oriented behaviors interspersed with temporally increasing bouts of immobility. A 4-minute test session was scored by a trained observer who was unaware of the treatment and/or genotype. The parameter recorded was the number of seconds spent immobile. All experimental testing sessions were conducted between 9:00 am and 2:00 pm, with animals randomly assigned to treatment conditions and tested in counterbalanced order. In subchronic treatment, citalopram was injected i.p. at a dose of 10mg/kg at 23.5, 5, and 1 hour for a total of 3 doses (30mg/kg/24h) before exposure to TST. Control animals were injected with 0.9% saline following the same schedule.

### Surgical Procedures

Mice anesthetized with 5% isoflurane in humidified O_2_ and positioned in a stereotaxic frame (Stellar; Stoelting Co., Wood Dale, IL) were implanted with one guide cannula (CMA/7, CMA Microdialysis). Each mouse was implanted with one guide cannula according to the following coordinates from bregma (Paxinos and Franklin, 1997): cortex, AP=+2.1, L=-1.0, DV=-1.5; hippocampus, AP=-3.0, L=+3.0, DV=-1.8. A surgical screw served as an anchor, and the cannulae were fixed to the skull with acrylic dental cement.

### α-FMHis i.c.v. Infusion

Mice were anesthetized as above and placed on a stereotaxic frame (Kopf Instruments). A stainless-steel cannula (7mm in length, outer diameter 0.5mm, and inner diamter 0.25mm) was implanted in the lateral ventricle and fixed to the skull using dental cement. The following coordinates were used according to the mouse brain atlas (Franklin and Paxinos, 2007): AP -0.3; L ±1; DV -1. After 7 days of recovery, α-FMH was infused into ventricle. A stainless-steel injection micro-needle was connected through a polyethylene catheter to a 1000-µL Hamilton precision syringe and then lowered into the lateral cerebral ventricle (dorsoventral, DV 2.4mm). α-FMHis was delivered via an infusion pump (5 µL) within 5 minutes. After infusion, the needle was left in place for an additional minute.

### Microdialysis Experiments

Microdialysis was performed 48 hours after surgery, during which mice recovered from surgery, housed one per cage. The stylet was removed from the guide cannulae, and the microdialysis probes (CMA/7 7/2 Cuprofane; molecular mass cutoff 6000Da; CMA Microdialysis) were inserted; the dialyzing membrane protruded 2mm from the tip of the cannula. Probes were perfused with Ringer’s solution (147mM NaCl, 2.4mM CaCl_2_, and 4.0mM KCl, pH 7.0) at a flow rate of 1 µL/min by use of a microperfusion pump (Mod CMA/100; Carnegie Medicine, Stockholm, Sweden). Two hours after insertion of the microdialysis probes, when neurotransmitter release became stable, collection of 30-minute fractions was started. Spontaneous release was defined as the average value of the first three/four 30-minute fractions collected during 90/120 minutes of perfusion with Ringer’s solution before drug treatment. All subsequent fractions were expressed as percentage increase of this value. Drug addition to the perfusion fluid did not modify the pH of the medium. Histaminergic neurons are more active during wakefulness, their activity being lowest during quiet waking and highest during attentive waking (Takahashi et al., 2006). In the present study, the experiments were performed during the light phase, and histaminergic cell activity was presumably low. Accordingly, most of the mice (about 90%) were sleeping for the entire period preceding citalopram administration that produced weak signs of awakening but not of agitation or irritability.

### Determination of HA

To prevent degradation of histamine (HA), 1.5 µL of 5mM HCl was added to each sample. The dialysates were kept at -80°C until analysis. HA contents in the dialysates were determined by HPLC-fluorometry (Cenni et al., 2006). In brief, the column (Hypersil ODS, 3 µm, 2.1×100mm; Thermo Fisher Scientific, Waltham, MA) was eluted with 0.25M potassium dihydrogen phosphate containing 5% octanesulfonic acid (Sigma-Aldrich, St. Louis, MO) at a flow rate of 0.4mL/min. The eluate from the column was mixed first with 0.1% *o*-phthalaldehyde solution at a flow rate of 0.1mL/min and then to a solution containing 4M sodium hydroxide and 0.2M boric acid (flow rate, 0.137mL/min) to adjust the reaction mixture to pH 12.5. The reaction took place at 45°C. Then 17% orthophosphoric acid was added to the solution (flow rate, 0.137mL/min) to reach a final reaction mixture at pH 3. The fluorescent intensity was measured with a spectrofluorometer (series 1100; Agilent, Waldbronn, Germany) at 450nm with excitation at 360nm. The sensitivity limit was 10fmol and the signal/noise ratio was higher than 3. HA levels in the dialysate samples were calculated as fmol/30min. In α-FMHis–treated mice, HA levels were below the detection sensitivity limits of the apparatus.

### Determination of 5-HT, NA, and DA

The dialysates were kept at -80°C until analysis. HPLC was performed as described (Cassano et al., 2009). Briefly, the endogenous levels of dopamine (DA), noradrenaline (NA), and 5-HT were assayed by microbore HPLC using a SphereClone 150-mm×2-mm column (3-µm packing). Detection was accomplished with a Unijet cell (BAS) with a 6-mm-diameter glassy carbon electrode at +650 mV vs an Ag/AgCl reference electrode connected to an electrochemical amperometric detector (INTRO, Antec Leyden, Netherlands). The chromatographic conditions were: (1) a mobile phase composed of 85mM of sodium acetate, 0.34mM Ethylenediaminetetraacetic acid (EDTA), 15mM sodium chloride, 0.81mM of octanesulphonic acid sodium salt, 5% methanol (vol/vol), pH=4.85; (2) a rate flow of 220 µL/min; and (3) a total runtime of 65 minutes. For each analysis, a set of standards containing various concentrations of each compound (monoamines and metabolites) was prepared in the acid solution and was injected before, and at the end, of any one run to account for changes in chromatographic conditions during the run. The calibration curves were calculated by linear regression using the mean of the standard values before and after the run. The retention times of standards were used to identify peaks, and peak areas were used to quantify neurotransmitters levels, calculated as fmol/30min.

### Histology

The placement of microdialysis membranes was verified postmortem. Mice were overdosed with chloral hydrate and the brains removed and stored in 10% formalin for 10 days. Forty-micrometer sections were then sliced on a cryostat, mounted on gelatin-coated slides, and stained with cresyl violet for light microscopic observation. Data from mice in which the membranes were not correctly positioned were discarded (<5%).

### 8-OH-DPAT–Induced Hypothermia

Body temperature was measured with a digital thermometer (TH5 Thermalert monitoring thermometer, Physitemp Instruments) equipped with a probe for mice (RET-3) inserted 1.0cm into the rectum. All temperatures were measured at ambient temperature (23±1°C). Basal temperature was measured 30 minutes and immediately prior to the subcutaneous (s.c.) injection of 8-hydroxy-2-(di-n-propylamino)tetralin hydrobromide (8-OH-DPAT; 1mg/kg, dissolved in 0.1mL saline). Body temperature was recorded every 15 minutes for 1 hour after injection.

### Western-Blotting Analysis

Hippocampi were homogenized in 0.2mL ice-cold lysis buffer (50mM Tris, pH 7.5, 50mM NaCl, 10mM ethylene glycol tetraacetic acid, 5 mM Ethylenediaminetetraacetic acid, 2mM SodiumPyrophosphate, 4mM Para-Nitrophenylphosphate, 1mM Na_3_VO_4_, 1.1mM Phenyl-methyl-sulphonyl fluoride, 20 µg/µL leupeptin, 50 µg/µL aprotinin, 0.1% SDS) using a pestle, sonicated briefly, and centrifugated at 12000rpm at 4°C for 15 minutes. The supernatant was collected and protein concentration was determined by Pierce BSA (Thermo Scientific). Samples were diluted in a mix of lysis buffer and loading buffer 2× (50mM Tris, pH 6.8, 100mM DL-dithiotheitol (DTT), 10% glycerol, 1% bromophenol blue, and 2% sodium dodecyl sulphate (SDS)) and boiled for 10 minutes at 95°C. Aliquots containing 40 µg total proteins were separated on 8% (SDS)-polyacrylamide gel electrophoresis and transferred to polyvinylidene difluoride membranes (Immobilon Transfer Membranes, Millipore). Blots were blocked in Tris-buffered saline, pH 7.6, containing 0.1% of Tween 20 (TBS-T) and 5% skimmed milk (Bio-Rad Laboratories) for 2 hours at room temperature and then incubated overnight, on different days, with monoclonal antibodies against phospho-CREB (pCREB-Ser133) (1:1000, catalogue no. 9198) and CREB (1:1000, catalogue no. 9197, both from Cell Signaling Technology), which were dissolved in TBS-T with 5% bovine serum albumin. Membranes were then washed 3 times with TBS-T and incubated for 120 minutes at room temperature in TBS-T with 1% skimmed milk containing anti-rabbit peroxidase-conjugated secondary antibody (1:5000, catalogue no. 7074, Cell Signaling Technology). After washing in TBS-T 3 times, enhanced chemiluminescence reaction (Luminata Crescendo, Millipore) was used to visualize the peroxidase-coated bands. The bands were quantified by densitometry analysis using an ImageQuant 350 imager and ImageQuant TL software (Perkin Elmer). pCREB densities were divided by their respective CREB densities within each sample to obtain pCREB:CREB ratio values and were averaged for each treatment group.

### Statistical Analysis

All values are expressed as means ± SEM, and the number of animals used in each experiment is indicated. Data were analyzed using 1-way ANOVA and Neuman-Keuls multiple comparison test or Scheffe’s test, unless otherwise stated, using the software GraphPad Prism. The level of significance was set at *P*≤.05. Data of the microdialysis experiments were analysed using a 2-way ANOVA and Bonferroni’s test.

## Results

### Effects of Various Antidepressants in the TST

**Figure 1.**
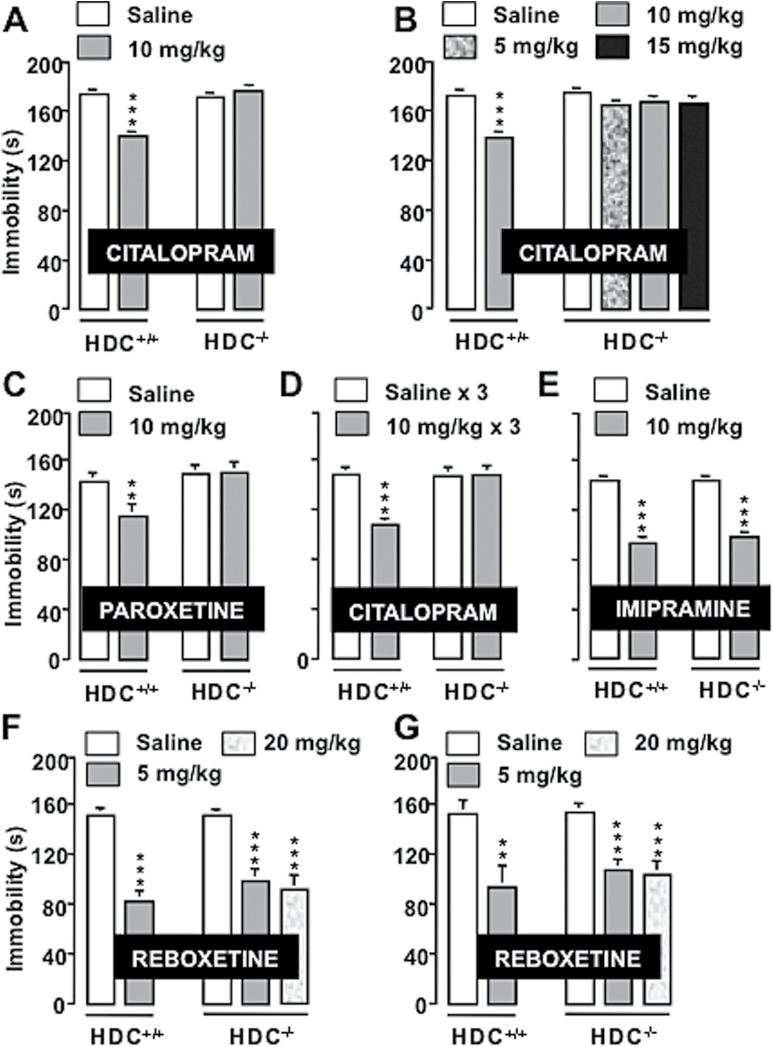
Behavioral effects of antidepressants in mice genetically deprived of histamine (HA). The selective serotonin reuptake inhibitors (SSRIs) citalopram (A, males; B, female) and paroxetine (C) reduced immobility time of HDC^+/+^ but not of HDC^-/-^ mice. Three citalopram injections within 24 hours reduced immobility time only in HDC^+/+^ mice (D). The tricyclic antidepressant (TCA) imipramine decreased immobility time of both genotypes (E). The Serotonin–norepinephrine reuptake inhibitor (SNRI) reboxetine reduced immobility time in males (F) and females (G) of both genotypes. Shown are means ± SEM of 8 to 18 mice/group. **P<.01, ***P<.001 vs saline-treated mice of the corresponding genotype (ANOVA and Neuman-Keuls multiple comparison test). Saline-treated mice of both genotypes and genders showed no differences in immobility in all experiments.

We first evaluated the effect of 2 SSRIs, citalopram and paroxetine (Bezchlibnyk-Butler et al., 2000), on the TST, a widely used paradigm for assessing antidepressant activity (Steru et al., 1985), in HDC^-/-^ mice and littermates. The TST is a common model of stress-induced behavioral depression, and all major classes of antidepressants effectively reduce immobility in this test, confirming its validity as a drug-screening paradigm (Cryan et al., 2005). There was no significant difference of immobility time between saline-treated HDC^+/+^ and HDC^-/-^ mice (Figure 1). Intraperitoneal administration of citalopram significantly decreased immobility of male HDC^+/+^ (1-way ANOVA and Neuman-Keuls multiple comparison test, F_3,54_=13.948; *P*<.0001; Figure 1a) and female (F_5,65_=6.1; *P*<.0001; Figure 1b) mice, whereas no significant effects were observed in HDC^-/-^ mice of either gender. Paroxetine reduced immobility of HDC^+/+^ (F_3,38_=7.824, *P*<.0001), whereas it was ineffective in HDC^-/-^ mice (Figure 1c). Different responses are reported after acute or repeated citalopram administration (Mombereau et al., 2010). In our paradigm, 3 administrations of citalopram during 24 hours significantly reduced immobility of HDC^+/+^ mice (F_3,44_=9.236, *P<*.0001) but not of their HDC^-/-^ littermates in a comparable fashion with single administrations (Figure 1d).

To test whether absence of response to SSRIs correlates with the selectivity to inhibit 5-HT reuptake, HDC^+/+^ and HDC^-/-^ mice received i.p. injections of imipramine or reboxetine, 2 antidepressants that block NA reuptake (Wong et al., 2000; Hajós et al., 2004). Imipramine significantly decreased immobility of both genotypes (F_3,44_=43.217, *P<*.0001; Figure 1e), as well as reboxetine (males: F_4,54_=12.172, *P<*.0001; Figure 1f; females: F_4,55_=7.199, *P<*.0001; Figure 1g) compared with saline-treated controls.

### Functional Integrity of the Serotonergic System

**Figure 2.**
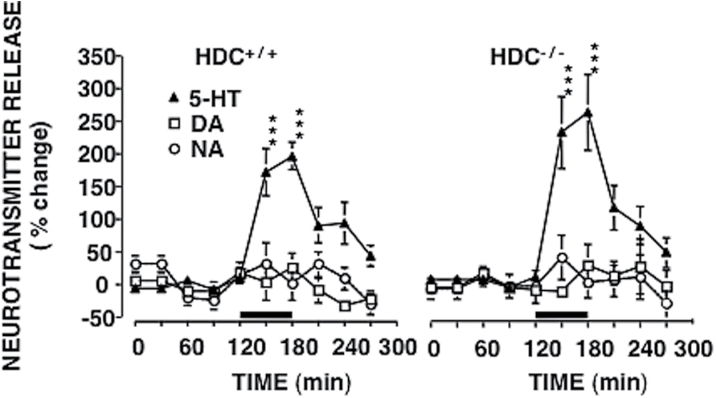
Effects of citalopram on hippocampal extraneuronal levels of 5-HT, dopamine (DA), and noradrenaline (NA) from hippocampi of HDC^+/+^ and HDC^-/-^ mice. (A) Neurotransmitter extraneuronal levels were measured in fractions collected every 30 minutes. Baseline values were determined from the average of 4 samples preceding citalopram infusion, which was begun at time 120 and terminated at time 180. No significant differences were observed between HDC^+/+^ and HDC^-/-^ mice. Citalopram significantly increased the extraneuronal levels of 5-HT in the hippocampus of both genotypes, but not of DA or NA. The mean basal extraneuronal level of 5-HT was 69±15fmol/30min in HDC^-/-^ mice (n=8) and 77±11fmol/30min in HDC^+/+^ mice (n=8). The mean of the DA basal extraneuronal level was 68±5fmol/30min in HDC^-/-^ mice (n=6) and 67±7fmol/30min in HDC^+/+^ mice (n=6). The mean of the NA extraneuronal level was 17±5fmol/30min in HDC^-/-^ mice (n=6) and 15±5fmol/30min in HDC^+/+^ mice (n=6). Represented are means ± SEM of 6 to 8 mice. ***P<.001 from “citalopram” and “cortical methysergide + citalopram” (2-way ANOVA and Bonferroni test).

**Figure 3.**
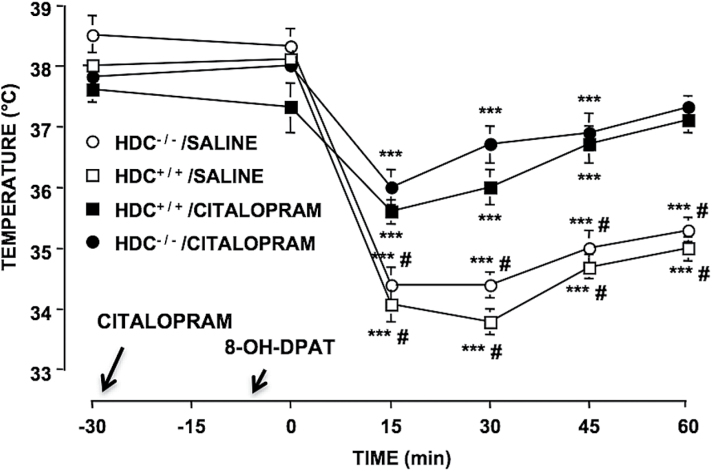
Effects of citalopram on hypothermic response induced by 8-Hydroxy-2-(di-n-propylamino)tetralin hydrobromide (8-OH-DPAT) in HDC^+/+^ and HDC^-/-^ mice. 8-OH-DPAT (1mg/kg, s.c.) similarly decreased body temperature in HDC^+/+^ and HDC^-/-^ mice. A single administration of citalopram 30 minutes prior to 8-OH-DPAT injection attenuated hypothermic response elicited by the prototypical agonist in both genotypes. Moreover, citalopram failed to affect basal temperature. Represented are means ± SEM of 6 to 7 mice. ***P<.001 from basal temperature of the same group; ^#^ *P<*.05, ^##^ *P<*.01 from saline of corresponding genotype (2-way ANOVA and Bonferroni test).

We checked the functional integrity of the serotonergic transmission in HDC^-/-^ mice performing in vivo microdialysis experiments. Perfusion of the dorsal hippocampus with 50 µM citalopram significantly increased 5-HT extraneuronal levels with similar magnitude in both HDC^+/+^ (F_9,70_=13.08; *P<*.0001) and HDC^-/-^ (F_9,70_=9.67; *P<*.0001) mice, leaving DA and NA release unchanged (Figure 2). Moreover, since desensitization of 5-HT_1A_ autoreceptors presumably plays a key role in the therapeutic action of SSRIs (Piñeyro and Blier, 1999), whereas their upregulation could be related to core symptoms of depression (Stockmeier et al., 1998), we checked the functional integrity of the serotonergic transmission in HDC^-/-^ mice also by using the hypothermic response to the 5-HT_1A_ agonist 8-OH-DPAT. 5-HT_1A_ autoreceptors in raphe neurons participate in the control of central serotonergic tone by mediating a negative feedback regulation of neuronal firing. 8-OH-DPAT–induced hypothermia appears related to activation of 5-HT_1A_ autoreceptors and decreased 5-HT release (Goodwin et al., 1985b) and hence provides a valid measure of the functional status of 5-HT_1A_ autoreceptors (Alexandre et al., 2006). Subcutaneous injections of 8-OH-DPAT induced significant decreases of body temperature that were equivalent in HDC^+/+^ and HDC^-/-^ mice (F_5,115_=57.06; *P<*.0001) (Figure 3). A single administration of citalopram 30 minutes prior to 8-OH-DPAT significantly attenuated 8-OH-DPAT–induced hypothermia in both genotypes (F_3,115_=14,85; *P<*.0001) without affecting basal temperature (Figure 3).

### Influence of Acute HA Depletion on Antidepressant Effects in the TST

**Figure 4.**
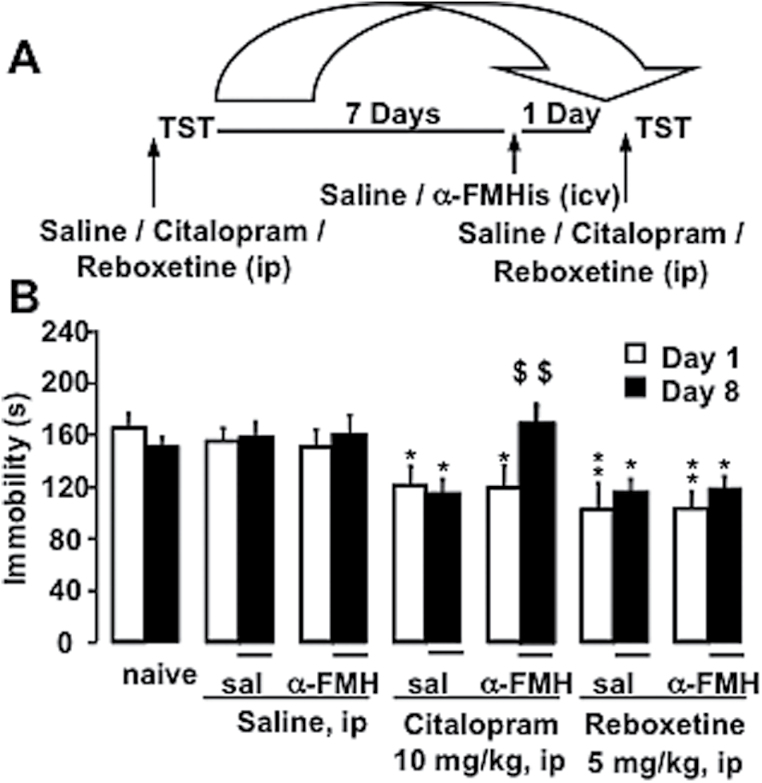
Behavioral effects of antidepressants in mice acutely deprived of brain histamine (HA). (A) Schematic representation of the protocol used to evaluate saline, citalopram, or reboxetine effects on the tail suspension test (TST) in CD-1 mice before and after treatment with alpha-fluoromethylhistidine (α-FMHis). (B) Effects of treatment with citalopram and reboxetine on immobility before and after treatment with a-FMHis. Animals were divided into 7 groups run simultaneously. All data were collated. Saline, citalopram, or reboxetine was given i.p. 30 minutes before testing on day (D)1 and D8. On D7, mice received i.c.v. injections of saline or a-FMHis. Naive mice received no treatment. Represented are means ± SEM of 7 to 11 mice. *P<.05, **P<.01 vs corresponding saline on D1 or D8, ^$$^ *P*<.01 vs same group D7 (ANOVA and Scheffe’s test).

**Figure 5.**
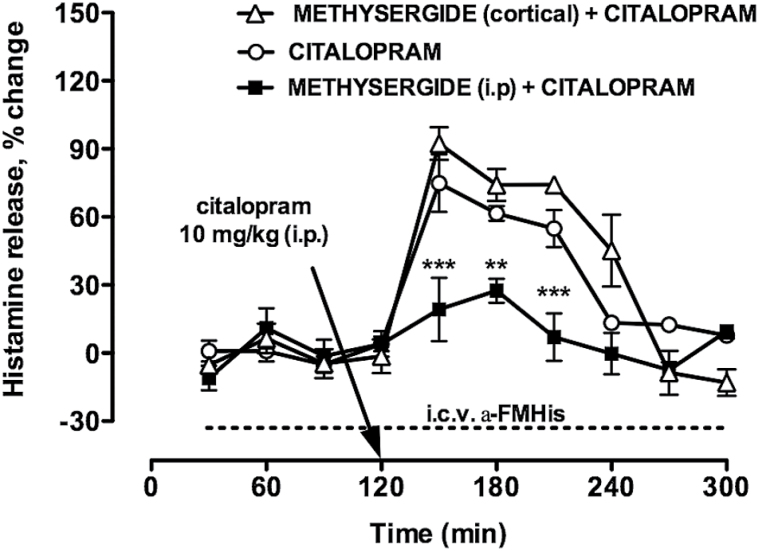
Effects of citalopram on cortical histamine (HA) extraneuronal levels of freely moving, CD1 mice. Citalopram significantly increased HA extraneuronal levels immediately after its administration (n=6). Systemic administration of methysergide prevented citalopram-elicited increase of HA extraneuronal levels (n=4), but not when administered in the cortex (n=3). The arrow indicates the time of citalopram administration. Baseline HA extraneuronal levels were not detectable in alpha-fluoromethylhistidine (α-FMHis)–treated mice. In these animals, HA extraneuronal levels were not detectable also during citalopram administration (dashed line; n=3). Represented are means ± SEM. ** *P<*.05, ***P<.001 vs “citalopram” and “methysergide (cortical) + citalopram”. Two-way ANOVA and Bonferroni’s test.

As the findings in HDC^-/-^ mice may be hindered by compensatory mechanisms, we investigated the effects of citalopram and reboxetine in CD-1 mice depleted of HA, by i.c.v. injection of α-FMHis, an irreversible HDC inhibitor, that completely suppressed spontaneous and citalopram-evoked HA cortical release (Figure 5). Effects on TST were examined before and after i.c.v. injection of α-FMHis in the same mice according to the protocol shown in Figure 4a. Mice were divided into 7 groups run simultaneously and exposed to TST on day 1 (D1) and day 8 (D8) after different treatment combinations. Naive mice received no treatment. On D1, 2 groups received i.p. saline injections, 2 groups citalopram, and 2 groups reboxetine 30 minutes before TST (Figure 4b). On D7, one saline group received an i.c.v. saline injection, the other an i.c.v. injection of α-FMHis. The same pattern was applied to mice treated with citalopram or reboxetine on D1 (Figure 4b). On D8 and 30 minutes before TST, D1 saline groups received saline i.p. injections, D1 citalopram-treated mice i.p. injections of citalopram, and D1 reboxetine-treated mice i.p. injections of reboxetine. ANOVA revealed a significant treatment effect (F_13,116_=22.101, *P<*.0001). Scheffe’s posthoc analysis showed that citalopram- or reboxetine-treated mice on D1 spent significantly less time immobile than corresponding control groups (saline-treated and naive mice on D1, *P<*.05-0.01) (Figure 4b). Mice responded to reboxetine in a similar fashion on D1 and D8 independently of saline or α-FMHis treatment on D7. However, mice receiving α-FMHis on D7 and citalopram on D8 spent significantly more time immobile than on D1 (*P<*.01) and more than those receiving i.c.v. saline on D7 (*P<*.01), indicating that they became insensitive to the behavioral effects of citalopram (Figure 4b).

### Functional Interactions between the Serotonergic and Histaminergic Systems

Prompted by our behavioral results, we investigated the functional interaction between the serotonergic and histaminergic systems. We first tested the effect of citalopram on HA extraneuronal levels in the cortex of freely moving CD1 mice. Citalopram significantly increased HA extraneuronal levels immediately after its administration, up to a peak value of 75±14% (F_9,59_=26.12; *P<*.0001) (Figure 5). Mean basal extraneuronal levels were 75±10fmol/30min (n=4). We then determined if endogenous 5-HT is involved in citalopram-evoked increase of HA extraneuronal levels. Methysergide, a 5-HT_2A/2c_ receptor antagonist (Martin and Sanders-Bush, 1982), given i.p. 30 minutes before citalopram fully antagonized citalopram-elicited increase of HA extraneuronal levels (Figure 5). The mean basal extraneuronal level of HA was 66±7fmol/30min (n=6). In another set of experiments, 10 µM methysergide was included in the cortical perfusion medium 30 minutes before citalopram and maintained for 120 minutes. Local administration of methysergide did not affect citalopram-elicited increase of HA extraneuronal levels (peak value of 92±7%; F_9,39_=26.08; *P<*.0001) (Figure 5). The mean extraneuronal level of HA was 72±11fmol/30min (n=3). Two-way ANOVA revealed a significant effect of treatment interactions (F_18,102_=5.1; *P<*.0001). Posthoc analysis revealed that systemic but not local treatment with methysergide significantly the citalopram-elicited increase of HA extraneuronal levels. In α-FMHis–treated mice, basal HA extraneuronal levels were below the detection sensitivity (Figure 5). In these animals, citalopram failed to increase HA extraneuronal levels above the detection sensitivity (Figure 5).

### CREB Phosphorylation in the Hippocampus of HDC^+/+^ and in HDC^-/-^ Mice

**Figure 6.**
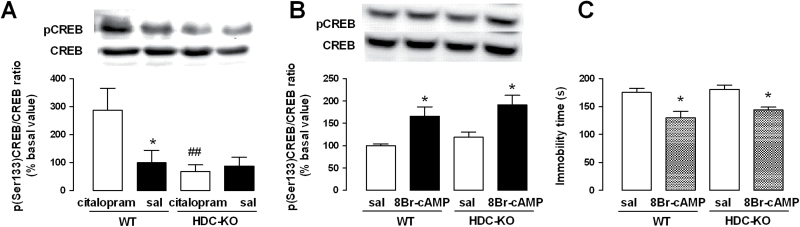
Citalopram-induced CREB phosporylation is absent in histamine (HA)-deprived mice. (A) Subchronic administrations of citalopram (3×10mg/kg, i.p. in 24 hours) increased the phosphor-CREB (pCREB)/CREB ratio in the hippocampus of HDC^+/+^ but not of HDC^-/-^ mice, as shown by Western-blot analysis. Shown are means ± SEM of 6 to 10 animals for each group; *P<.05, ##*P<*.01 vs citalopram-treated HDC^+/+^ mice. (B) Administration of 8-bromoadenosine 3’, 5’-cyclic monophosphate (8-Br-cAMP; i.c.v., 5 µg/5 µL 30 minutes prior to sacrifice) increased the pCREB/CREB ratio in the hippocampus of mice of both genotypes. Shown are means ± SEM of 4 to 5 mice per group (*P<.05, vs 8-Br-cAMP-treated mice, ANOVA, and Newman-Keuls post-hoc test). (C) Administration of 8-Br-cAMP significantly reduced immobility of mice of both genotypes in the tail suspension test (TST) (*P<.05 vs saline-treated mice; n=8–9/group).

Correlation between SSRIs administration and increases in CREB phosphorylation in the hippocampus supports CREB’s role in mediating antidepressant effects and suggests the hippocampus as an important neural substrate mediating antidepressant responses (Berton and Nestler, 2006b; Blendy, 2006; Mombereau et al., 2010). Three injections of citalopram during 24 hours, sufficient to induce long-lasting adaptations produced by chronic administration of antidepressants (Conti et al., 2002; Gur et al., 2007), significantly increased pCREB in the hippocampus of HDC^+/+^ but not of HDC^-/-^ mice compared with saline-treated animals of the same genotype (F_3,28_=4.89, *P<.*05) (Figure 6a). Phosphorylation of CREB is initiated by cAMP-dependent protein kinase. To investigate the integrity of this pathway, we treated HDC^+/+^ and HDC^-/-^ mice with 8-bromoadenosine 3’, 5’-cyclic monophosphate (8-Br-cAMP), a long-acting activator of protein kinase. I.c.v. administration of 8-Br-cAMP (5 µg/5 µL, 30 minutes prior to sacrifice) increased pCREB in the hippocampus of mice of both genotypes (F_3,15_=6.301; *P<.*05) (Figure 6b). Furthermore, 8-Br-cAMP significantly reduced the immobility time of mice of both genotypes (F_3,32_=9.896, *P<.*05) compared with saline-injected controls in the TST (Figure 6c). These results clearly indicate that CREB intracellular machinery is not impaired in HDC^-/-^ mice.

## Discussion

The present study provides strong evidence that intact HA neurotransmission is required specifically for the SSRIs citalopram and paroxetine to reduce immobility in the TST. All antidepressants effectively reduce immobility in this test (Lucki et al., 2001). Antidepressants of other classes, such as imipramine (tricyclic) or reboxetine (selective NA reuptake inhibitor), were equally effective at reducing immobility in mice with or without HA. Low levels of 5-HT may cause insensitivity to SSRIs in the TST (O’Leary et al., 2007). This is not the case for HDC^-/-^ mice, since citalopram induced comparable increases of 5-HT extraneuronal levels in the hippocampus of HDC^-/-^ and HDC^+/+^ mice. In addition, citalopram application did not increase DA or NA extraneuronal levels, confirming its selectivity for 5-HT transporters (Millan et al., 2001). Desensitization of the 5-HT_1A_ receptor is thought to mediate at least in part the therapeutic efficacy of SSRIs (Gray et al., 2013). The normal functioning of this receptor can be assessed by measuring the hypothermic response elicited by 8-OH-DPAT, a 5-HT_1A_ agonist (Hjorth, 1985; Seletti et al., 1995; Evrard et al., 2002). 8-OH-DPAT–induced hypothermia is presumably related to a drop in extracellular levels of 5-HT mediated by somatodendritic 5-HT_1A_ autoreceptors in the raphe (Goodwin et al., 1985a). The hypothermic response to 8-OH-DPAT was equivalent in HDC^-/-^ and HDC^+/+^ mice, suggesting a normal signal efficiency of 5-HT_1A_ receptors in HDC^-/-^ mice. Furthermore, a single administration of citalopram produced an equivalent attenuation of the hypothermic response to 8-OH-DPAT in both genotypes. These findings clearly rule out the possibility that HDC^-/-^ mice are insensitive to SSRIs because of serotonergic system modifications. Nonetheless, compensatory mechanisms in HDC^-/-^ mice may affect behavioral responses to SSRIs. In the TST test though, mice acutely deprived of brain HA lost their sensitivity to citalopram but not to reboxetine, supporting our view that insensitivity to SSRIs is directly consequent to the lack of HA.

**Figure 7.**
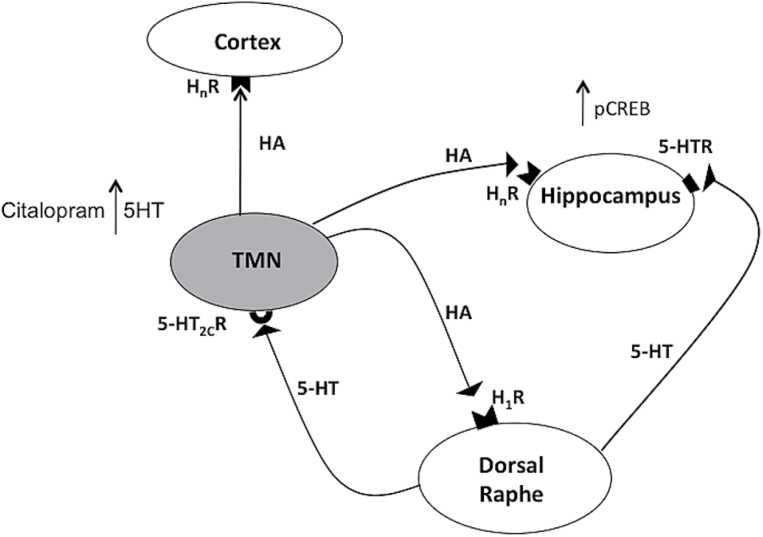
Schematic drawing illustrating the putative interactions between the serotonergic and histaminergic systems. Histamine (HA) neurons are localized exclusively in the tuberomamillary nucleus (TMN) of the posterior hypothalamus. The TMN receives dense serotonergic projections from the raphe nuclei (Ericson et al., 1989), and 5-HT depolarizes HA neurons by activating 5-HT_2C_ receptors (Eriksson et al., 2001). In turn, activation of HA H_1_ receptor (H_1_R) on dorsal raphe neurons increases the serotonergic neuron firing rate (Bárbara et al., 2002; Brown et al., 2002). The disruption of this loop in HA-deprived mice supposedly prevents CREB phosphorylation in the hippocampus and is responsible for the inefficacy of selective serotonin reuptake inhibitors (SSRIs) in the tail suspension test (TST). Blockade of 5-HT_2c_ receptors in the TMN inhibits HA release in the cortex. H_n_R, histamine receptors.

As brain HA signaling seems to be involved in the acute and subchronic effects of citalopram, we expected citalopram to influence HA extraneuronal levels. Indeed, systemic administration of citalopram at the same dose effective in the TST enhanced cortical HA extraneuronal levels. HA increase elicited by citalopram was antagonized by systemic but not intra-cortical administration of methysergide, thus suggesting the involvement of endogenous 5-HT. The lack of effect following intra-cortical administration of methysergide excludes the involvement of cortical 5-HT_2A/2C_ receptors. It was previously reported that the TMN receives dense serotonergic projections from the raphe nuclei (Ericson et al., 1989) and that 5-HT depolarizes directly HA neurons by activating 5-HT_2C_ receptors (Eriksson et al., 2001). Taken together, these results suggest that SSRIs increase extracellular levels of endogenous 5-HT in the TMN, which in turn impacts 5-HT_2C_ receptors localized on HA neurons, enhances their firing rate, and consequently augments HA release in the cortex (Figure 7). In turn, HA acts on H_1_-receptors in the raphe and increases serotonergic neurons’ firing rate (Bárbara et al., 2002; Brown et al., 2002) (Figure 7). We believe that disruption of this loop in HA-deprived mice is at least in part responsible for the inefficacy of SSRI in the TST. Our results also indicate that HA signaling is necessary for citalopram to trigger CREB phosphorylation, as repeated administrations of citalopram significantly increased pCREB in the hippocampus of HDC^+/+^ but not of HDC^-/-^ mice (Figure 7). CREB phosphorylation is one of the molecular mechanisms implicated in the efficacy of SSRI treatment (Carlezon et al., 2005) and is required for TST response to citalopram, as this is completely abolished in CREB-deficient mice (Gur et al., 2007).

The probability of achieving and sustaining symptomatic remission in major depressive disorder with first-line pharmacotherapy is approximately 30% (McIntyre, 2010); therefore, identifying predictors of response is a priority, as there are no biomarkers that can reliably predict treatment efficacy. The present findings demonstrate that histaminergic neurotransmission is indispensable for behavioral and neurochemical responses to acute administration of SSRIs. Evidence of the effects of long-term treatments would be more convincing in terms of possible clinical relevance. Indeed, SSRIs quickly inhibit the serotonin transporter yet begin to exert an antidepressant response in patients during the course of 2 to 3 weeks of administration. Nevertheless, we may speculate that neuronal HA should be increased when SSRIs are taken. Single nucleotide polymorphism of the HA H_1_ receptor gene was found to play a role in bipolar disorder, as it was significantly associated with improvements following olanzapine and fluoxetine treatment (Perlis et al., 2010). In addition, functional mutation in the HDC gene resulting in deficits of the histaminergic neuronal system has been linked to the mechanism and modulation of Tourette’s syndrome and tics (Ercan-Sencicek et al., 2010; Castellan-Baldan et al., 2014). Similar genetic variations in the population may contribute to individual differences in antidepressant response and may prove good predictors of more effective treatments.

## Interest Statement

None.
